# Effect of pulse phase duration on forward masking and spread of excitation in cochlear implant listeners

**DOI:** 10.1371/journal.pone.0236179

**Published:** 2020-07-20

**Authors:** Ning Zhou, Zhen Zhu, Lixue Dong, John J. Galvin

**Affiliations:** 1 Department of Communication Sciences and Disorders, East Carolina University, Greenville, North Carolina, United States of America; 2 Department of Engineering, East Carolina University, Greenville, North Carolina, United States of America; 3 House Ear Institute, Los Angeles, California, United States of America; University College London, UNITED KINGDOM

## Abstract

Previous cochlear implant (CI) research has shown that at a pulse train with a long pulse phase duration (PPD) requires less current but greater charge to obtain the same loudness as a pulse train with a short PPD. This might result in different excitation patterns between long and short PPDs. At equal loudness, long PPDs might produce greater masking due to greater charge. However, because they require less current, long PPDs may produce a smaller spatial spread of excitation (SOE) compared to short PPDs by evoking a greater neural firing probability within the relatively small current field. To investigate the effects of PPD on excitation patterns, overall masking and SOE were compared for equally loud stimuli with short or long PPD in 10 adult CI ears. Forward masking patterns were measured at relatively soft, medium, and loud presentation levels. Threshold shifts were calculated in terms of percent dynamic range (DR) of the probe. The area under the curve (AUC) of the masking functions was significantly larger for the long PPD than for the short PPD masker. The difference in AUC was proportional to the difference in charge between the short and long PPD maskers. To estimate SOE, the masking patterns were first normalized to the peak masking, and then AUC was calculated. SOE was significantly larger for the short PPD than for the long PPD masker. Thus, at equal loudness, long PPDs produced greater overall masking (possibly due to greater charge) but less SOE (possibly due to less current spread) than did short PPDs. The effect of the interaction between masking and SOE by long PPD stimulation remains to be tested.

## Introduction

Most modern cochlear implant (CI) devices encode intensity by adjusting the pulse amplitude (PA) with a fixed pulse phase duration (PPD). In most CI systems, the PPD is typically short (≤25 μs/phase), allowing for high stimulation rates and rapid loudness growth with increasing PA. However, some CI devices currently encode intensity by varying PPD with a fixed PA (e.g., Oticon). In clinical practice, PPD may be increased beyond default values to reduce facial nerve stimulation, to accommodate patients with auditory neuropathy spectrum disorders, or to remain within voltage compliance [1]. However, perceptual differences between long and short PPDs are not well understood. In the present study, we examined the differences in forward masking and spatial spread of neural excitation (SOE) produced by short and long PPDs. The results may provide insights into optimizing PPD for individual patients in clinical fitting.

In CIs, the electric charge of a pulse is the product of PA and PPD. However, PA and PPD values do not trade-off linearly. Previous research has shown that equal charge does not correspond to equal loudness [2–5]. To maintain equal loudness, a stimulus with a relatively long PPD requires greater charge than does a stimulus with a relatively short PPD. Loudness also grows more slowly when increasing PA with a long PPD than a short PPD. This has been demonstrated psychophysically [2], physiologically [6], as well as in auditory nerve modeling [7]. The fact that short PPDs produce more efficient excitation than long PPDs reflects the “leakiness” of the auditory nerve. If the neural membrane could perfectly integrate charge over the entire PPD, threshold levels would be expected to reduce by 6 dB for every doubling of PPD. Psychophysical studies have reported slopes significantly less than 6 dB/doubling [8] and the slopes were even shallower for single auditory fiber recordings [9]. The efficiency of charge integration of the auditory nerve depends on a number of factors, including initiation site of action potential, the number of ion channels available for integration, and myelination [10]. The peripheral processes have a longer time constant for integration than do the central axons, but they are the first to degenerate with hearing loss. A secondary loss will result in a reduced number of auditory neurons, which reduces the number of ion channels for integration. Consistent with the idea that charge integration would worsen with degeneration, results of our parallel studies have shown that CI users with a longer duration of hearing loss before implantation exhibited a much larger dynamic ranges (DRs) with increasing PPD than with increasing PA [5]. Such “charge integration efficiency” may also vary across stimulation sites, consistent with the idea that pathology is uneven along the tonotopic axis [11].

While greater charge may be required for a long PPD to maintain loudness relative to a short PPD [2–5], it is unclear whether this greater charge would also produce greater masking. A stimulus produces forward masking when it induces neural adaptation and reduces the post-stimulus excitability. It is well established that neural adaptation is more likely to occur with rigorous and/or repetitive stimulation, which could result from using high PA or high stimulation rates [12–14]. One factor underlying the declining neural excitability over time is the depletion of neurotransmitters in synapses. This will reduce the neurons’ response to subsequent stimulation, thus producing forward masking. The first aim of the study was to examine whether sustained charging on the neural membrane with long PPDs would have similar effects on neural fatigue as observed with repetitive stimulation. To address this, we compared the amount of forward masking produced by equally loud maskers with relatively short or long PPDs. Since long PPDs require less PA, the lower PA might offset the effects of long PPDs on neural adaptation. However, if neural adaptation depends on the total charge exerted on the neural membrane, long PPDs would produce more masking than would short PPDs, for equally loud maskers. It is also possible that equally loud maskers with a short or long PPD would produce the same amount of masking, if loudness (the masker-induced neural activity) is the governing factor.

Even if the greater charge associated with longer PPDs produces greater masking, it is unclear whether the relative spread of excitation (SOE) would differ between equally loud short PPD and long PPD maskers. In this study, SOE, is defined as the rate of masking decay as a function of masker-probe electrode separation and represents the place specificity of neural excitation. The spatial decay can be quantified by calculating the area under the curve (AUC) of the masking function after normalizing to the peak masking. Note that a stimulus can produce a small amount of masking but a relatively large SOE. This would be the case where the masking functions are flat but have smaller absolute threshold shifts. Factors that contribute to SOE (e.g., loss of peripheral processes, neural density, electrode placement, etc.) [15] have been extensively studied using fixed, short PPDs. Much research effort has been made towards developing methods to reduce SOE and improve the place specificity of the spectral information. Such efforts include increasing the space between activated electrodes by removing channels [16–18], lowering the stimulation rate [19], and “current focusing” to spatially restrict current spread [20–25]. Current focusing via multipolar stimulation has shown only marginal improvements in SOE, partly due to the higher current levels needed to achieve sufficient loudness. The putative advantage of current focusing also depends on the number and excitability of neurons responding to the active electrode, which in turn depends on neural health and electrode location. DeVries and Arenberg [26] proposed that current focusing might be more effective if applied only to electrodes with greater distance from the modiolus and/or poor spatial tuning; however, speech perception data showed no advantage with the proposed current focusing over standard monopolar stimulation. Recent studies have also explored the efficacy of using asymmetric pseudo-monophasic pulses to reduce the side lobes to remove the undesired side effects with multipolar stimulation [27, 28]. These efforts have led to moderate success, but with high individual variability.

As noted above, long PPDs require less current (PA) to maintain loudness relative to short PPDs. If the lower PA with a long PPD results in a smaller current field, the same loudness should come from a smaller neural population operating at a higher firing probability, compared to a high PA/short PPD stimulus. This suggests that equally loud short and long PPD stimuli may excite different neural populations. This idea is supported by results from McKay and McDermott [29], who proposed that if equally loud short PPD and long PPD stimuli excite different neural populations, they should be discriminable. Some CI listeners in the study were able to do so, and participants who were able to consistently discriminate between short and long PPDs also reported that the stimuli differed in pitch. Nonetheless, possible differences in SOE between equally loud short PPD and long PPD stimuli have yet to be explicitly studied. There is indirect evidence showing that temporal information across widely spaced electrodes can be better integrated with long PPDs than with short PPDs [29]. This suggests that the long PPD produced a wider SOE than did the short PPD. However, the pattern was observed only in three participants, and other factors may have contributed to the integration of temporal information across electrodes. In the second part of this study, we explicitly compared SOE between equally loud stimuli with short or long PPDs. We hypothesized that the reduced current spread associated with long PPDs would produce less SOE (activating a smaller neural population) than would short PPDs.

In this study, forward masking patterns were measured in CI listeners for equally loud maskers with a short or long PPD at relatively soft, medium, and loud presentation levels. The total amount of masking and the rate of spatial decay of the masking (SOE) produced by the two maskers were compared.

## Materials and methods

### Participants and hardware

Ten CI ears (3 bilateral CI users, 4 unilateral CI users) were tested in this experiment. All participants except for bilateral CI user S16 were post-lingual users of Cochlear© devices (Cochlear Corporation, Englewood, CO); all had participated in a previous related study [5]. The mean age at testing was 67.8 years, the mean duration of deafness was 12.4 years, and the mean CI experience was 9.7 years. Demographic information for participants and test ears is shown in Table 1. All subjects provided written informed consent before taking part in the study. This study was approved by the East Carolina University institutional review board (UMCIRB 13–001783).

**Table 1:** Demographic information for CI test ears.

Test ear	Gender	Implant	Processor	Age test (yrs)	Dur Deafness (yrs)	CI exp (yrs)	Etiology
S1L	M	CI24 (CS)	CP910	79.3	0.1	16.2	Hereditary
S1R	M	CI24RE (CA)	CP810	79.3	6	10.2	Hereditary
S10L	F	CI24R (CS)	CP1000	68	0.8	17.1	Hereditary
S10R	F	CI24RE (CA)	CP1000	68	12.4	5.4	Hereditary
S16L	M	CI24RE (CA)	CP810	56.6	45.1	11.5	Hereditary
S16R	M	CI24RE (CA)	CP810	56.6	47	9.6	Hereditary
S4L	F	CI24RE (CA)	CP810	59.1	4.6	6.7	Hereditary
S18L	F	CI422	CP920	66.3	3.6	3.6	Hereditary
S19L	F	CI24RE (CA)	CP1000	71.5	4.3	10.9	Hereditary
S22R	F	CI24RE (CA)	CP900	73.4	0.4	5.8	Nerve damage
AVE				67.8	12.4	9.7	

Age test = age at testing; Dur Deafness = duration of deafness before CI; CI exp = CI device experience.

All stimuli were biphasic pulse trains (MP1+2 stimulation mode); the stimulation rate was 1000 pulses per second (pps) and the interphase gap was 8 μs. Masker stimuli were presented on Electrode (El) 11, with a 300-ms duration. Probe stimuli were presented to Els 8, 9, 10, 11, 12, 13, or 14, with a 20-ms duration. The masker-probe interval was 10 ms. The stimuli were controlled by MATLAB program interfacing with the NIC II research software. The subjects used a Nucleus^®^ Freedom processor (Cochlear Corporation, Englewood, CO) processor for all psychophysical testing. Using a research interface allowed explicit control of all stimulation parameters, which is not possible with the clinical speech processor.

### Masker levels

For the maskers, the DR was estimated between threshold and maximum acceptable loudness. Thresholds were measured using a method of adjustment and maximum acceptable loudness was measured using a method of limits [30]. For the short PPD maskers, the PPD was fixed at 25 μs/phase and the PA was increased until the stimulus was clearly audible. Participants were then instructed to adjust the PA until the stimulus was barely audible, first in steps of 5 clinical units (CUs), then in smaller steps of 1 CU. After obtaining threshold, the PA was increased in 5 CU steps until achieving maximum acceptable loudness. For the long PPD maskers, the threshold was the same as for the short PPD masker (i.e., 25 μs/phase with the PA measured for the short PPD masker described above). To obtain maximum acceptable loudness, the PA was fixed at threshold level and the PPD was increased in 5 and 1 μs/phase steps until achieving maximum acceptable loudness. Thus, the short PPD and long PPD maskers both had the same threshold. More details for the DR estimation procedures can be found in Zhou et al. [5].

For each probe electrode, the unmasked thresholds were estimated using a 3-alternative, forced-choice (3AFC) adaptive procedure (2-down, 1-up), converging on 70.7% correct on the psychometric function [31]. The PPD was fixed at 100 μs/phase. During threshold testing, the target was randomly assigned to one of the three intervals; the participant was asked to click on the interval that contained the target. The PA of the target was adjusted according to the correctness of response. The initial step size was 10 CUs and the step size was progressively reduced to 5, 2, and 1 CU. The PA at the last 6 reversal points was averaged as the threshold for the test run. At least two test runs were completed for each probe electrode, and the average across test runs was calculated as the probe threshold. The maximum acceptable loudness for each probe electrode was estimated using the method of limits [30]; the PA was slowly increased until achieving maximum acceptable loudness. The difference between threshold and maximum acceptable loudness was calculated to be the probe DR. Probe thresholds, maximum acceptable loudness levels, and DRs are listed in S1 Appendix.

The short and long PPD maskers were loudness-balanced at relatively soft, medium, and loud presentation levels using a method of adjustment [30]. The long PPD maskers served as references, and were presented at 30%, 50%, or 70% DR. During loudness balancing, the long (reference) and short PPD maskers were repeatedly presented in sequence. The PA of the short PPD masker was adjusted until achieving equal loudness as the long PPD reference; this procedure was repeated two times. Table 2 shows the percent DR for the short PPD maskers on a linear current amplitude scale (μA) and for the long PPD masker on a linear time scale (μs/ph), as well as the corresponding charge (nC).

**Table 2:** Percent DR, PPD values (μs/ph), PA values (μA), and charge values (nC) for equally loud long and short PPD maskers at the soft, medium, and loud masker levels.

Test ear	Masker level	long PPD				short PPD			
		% DR	μs/ph	μA	nC	% DR	μs/ph	μA	nC
S1L	Soft	30	49	277	13.6	37	25	485	12.1
	Medium	50	65	277	18.0	57	25	602	15.1
	Loud	70	81	277	22.4	74	25	695	17.4
S1R	Soft	30	44	282	12.4	37	25	469	11.7
	Medium	50	56	282	15.8	58	25	573	14.3
	Loud	70	69	282	19.5	73	25	651	16.3
S10L	Soft	30	35	367	12.8	51	25	600	15.0
	Medium	50	41	367	15.0	67	25	677	16.9
	Loud	70	48	367	17.6	96	25	811	20.3
S10R	Soft	30	65	136	8.8	37	25	355	8.9
	Medium	50	91	136	12.4	58	25	448	11.2
	Loud	70	117	136	15.9	73	25	561	14.0
S16L	Soft	30	77	253	19.5	56	25	639	16.0
	Medium	50	112	253	28.3	77	25	787	19.7
	Loud	70	146	253	36.9	89	25	869	21.7
S16R	Soft	30	73	263	19.2	52	25	713	17.8
	Medium	50	105	263	27.6	66	25	837	20.9
	Loud	70	137	263	36.0	77	25	930	23.3
S4L	Soft	30	55	126	6.9	36	25	254	6.4
	Medium	50	75	126	9.5	56	25	326	8.2
	Loud	70	95	126	12.0	75	25	395	9.9
S18L	Soft	30	73	135	9.9	38	25	350	8.8
	Medium	50	105	135	14.2	65	25	498	12.5
	Loud	70	137	135	18.5	82	25	594	14.9
S19L	Soft	30	52	117	6.1	42	25	227	5.7
	Medium	50	71	117	8.3	71	25	301	7.5
	Loud	70	89	117	10.4	88	25	345	8.6
S22R	Soft	30	52	125	6.5	39	25	246	6.2
	Medium	50	71	125	8.9	65	25	327	8.2
	Loud	70	89	125	11.1	94	25	418	10.5

### Forward masking procedure

Masked thresholds were adaptively measured at each probe electrode location for each masker at each presentation level using a 3AFC procedure. During each trial, the masker was presented in all three intervals and the probe was randomly presented in one of the three intervals. The listener responded by clicking on the interval in which the probe was heard. The PA of the probe started at 50% DR and was adjusted using a 2-down 1-up rule according to the correctness of response. The step sizes were the same as those used to measure unmasked thresholds (see above). The final 6 reversals in PA were averaged as the masked threshold for the test run. At least two test runs were completed for each probe and for each masker type (short or long PPD) and for each masker level. For each condition, the average across test runs was calculated as the masked threshold. In cases where thresholds differed by more than 2 dB, a third run was performed and the threshold was averaged across the runs with the closest thresholds. The threshold shift (masked—unmasked probe thresholds) was quantified in terms of percent DR of the probe as in McKay [32], and the calculation was in linear units (threshold shift in μA divided by DR in μA). Quantifying threshold shifts in percent DR (rather than in dB) allowed for better comparison of threshold shifts across stimulation sites. The AUC was calculated for the raw threshold shift data (in percent DR) to quantify the amount of masking produced by each masker for the three presentation levels; larger values indicated greater masking. AUC was calculated as the sum of the averaged masked threshold shifts between adjacent probe electrodes; larger values indicated greater overall masking. To estimate SOE, the masking functions were first normalized to the peak masking, and then AUC was re-calculated; larger values indicated broader relative spread.

## Results

**Fig 1:**
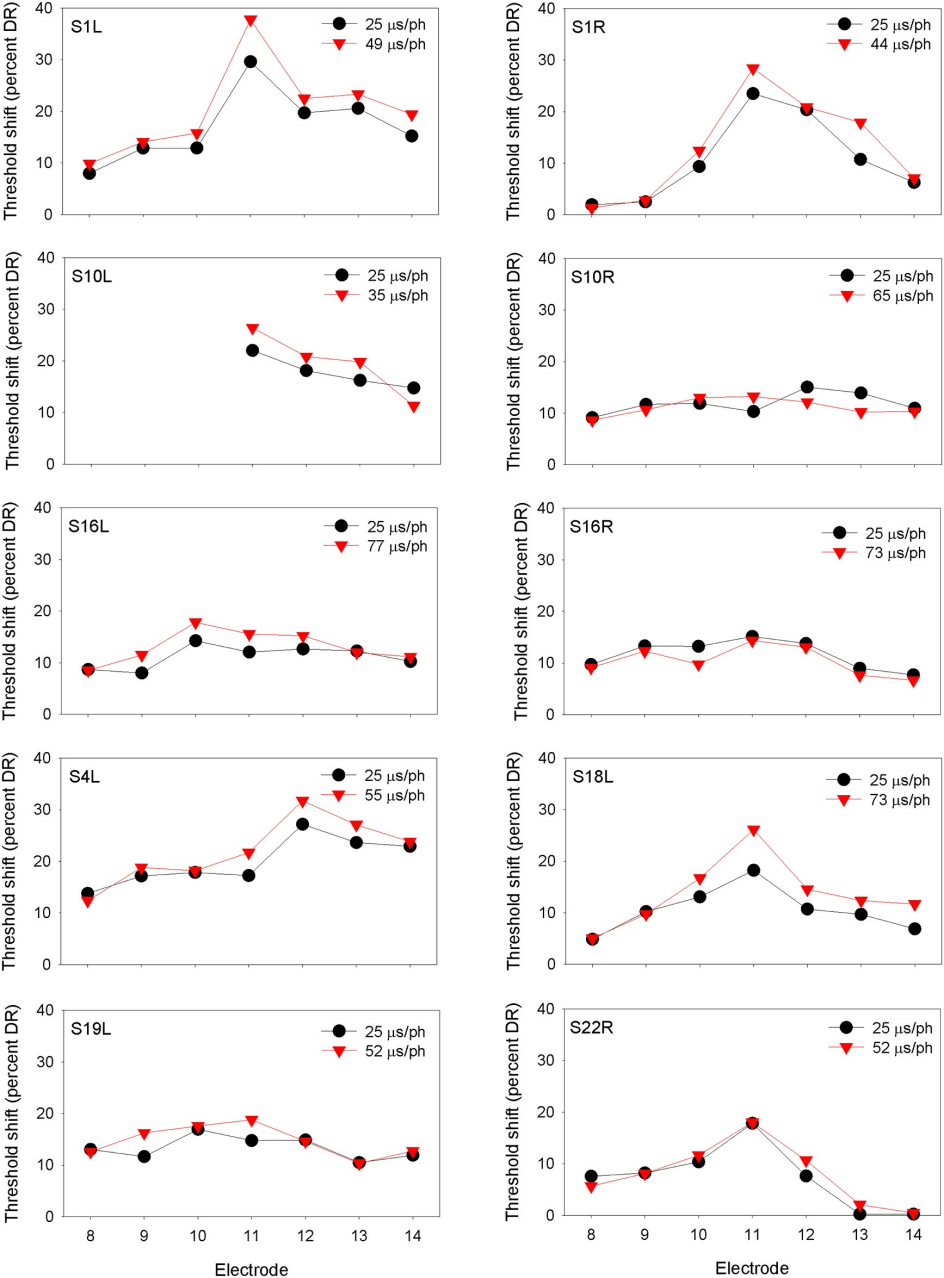
Threshold shift (in percent DR) at the soft presentation level for equally loud short PPD (black circles) and long PPD maskers (red triangles), as a function of probe electrode location. The PPDs of the maskers are shown in each panel.

**Fig 2:**
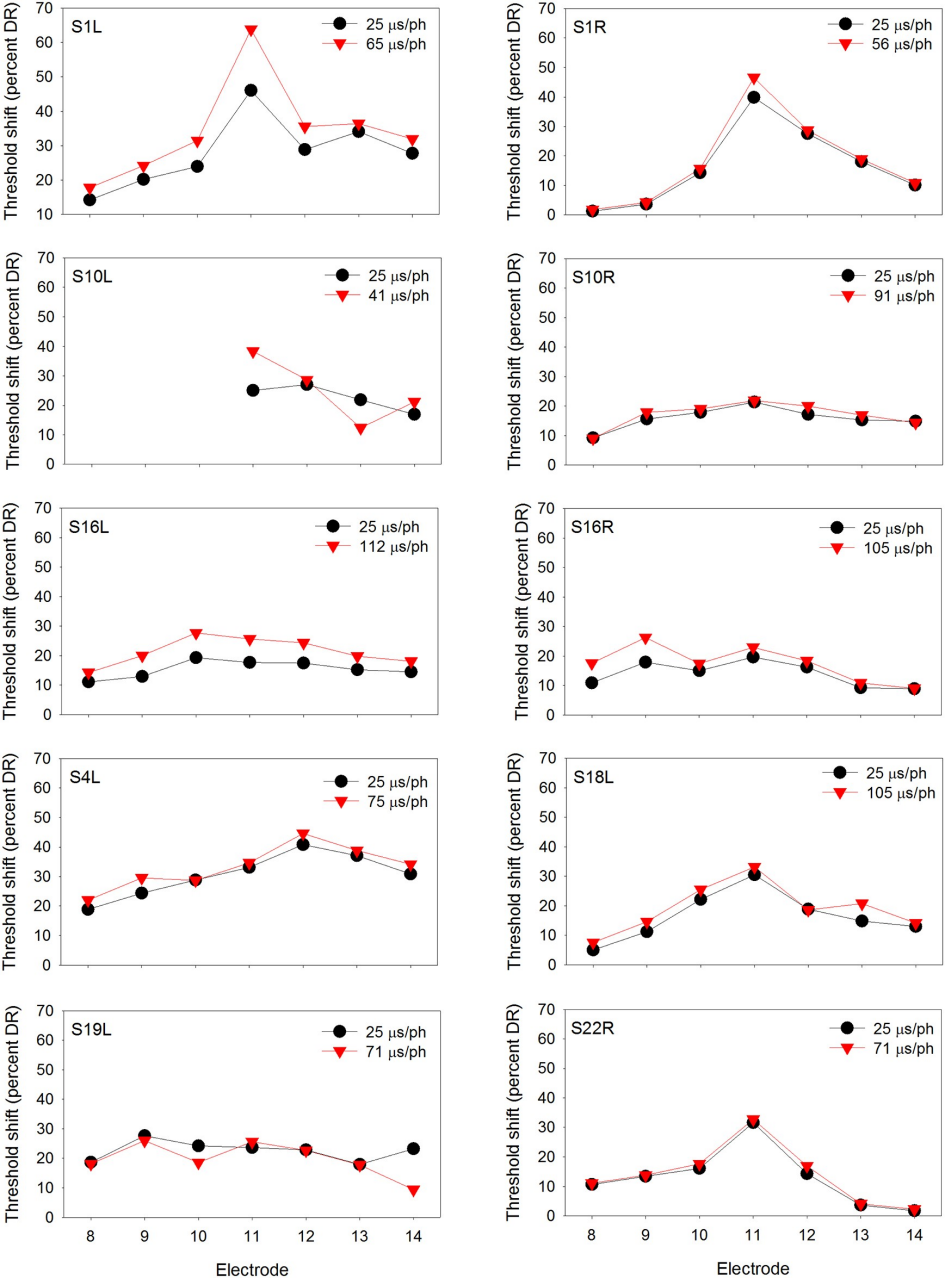
Same as Fig 1, but at the medium presentation level.

**Fig 3:**
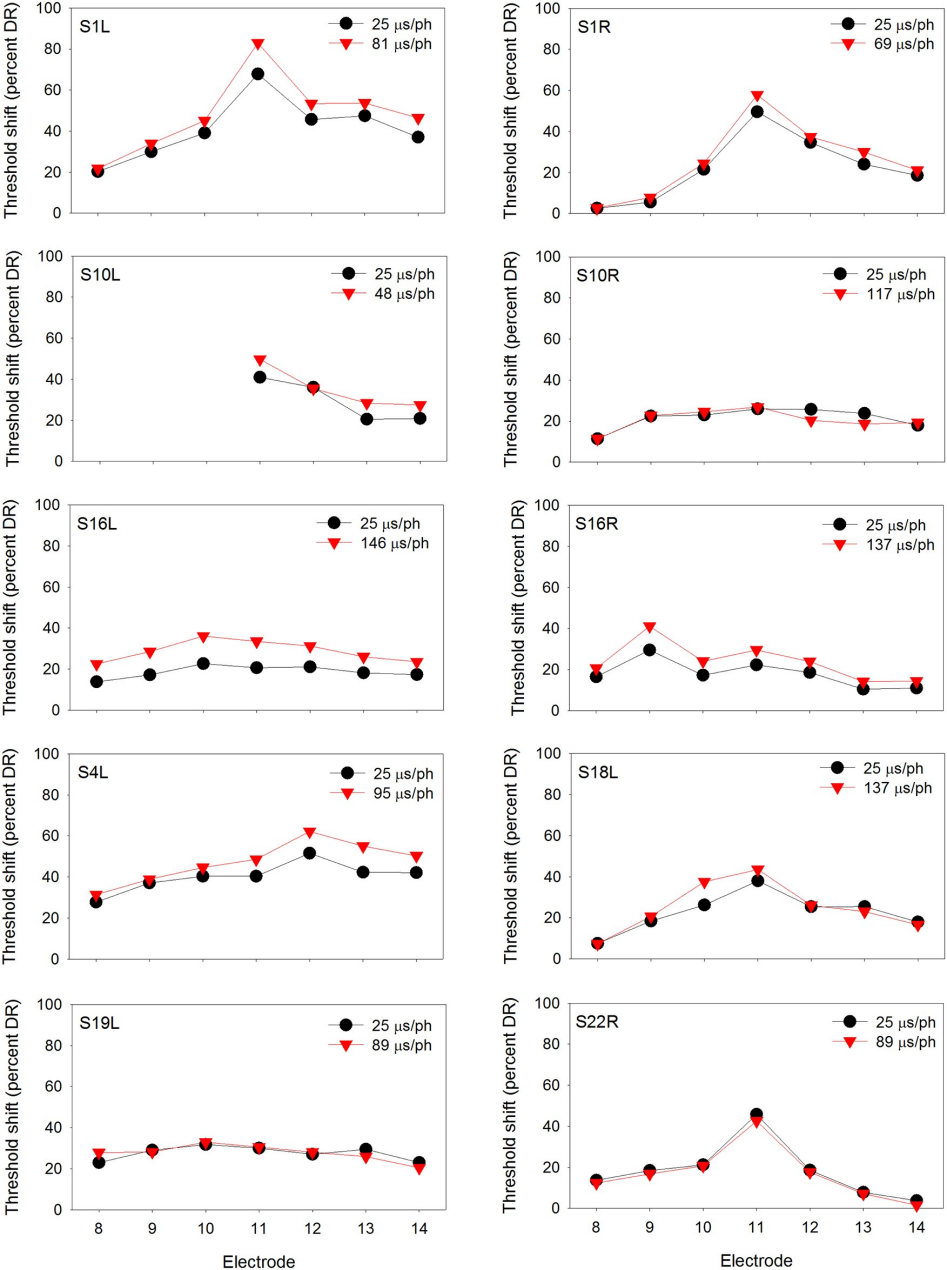
Same as Figs 1 and 2, but at the loud presentation level.

At equal loudness, the long PPD maskers contained significantly greater charge than the short PPD maskers [t (29) = 3.45, p = 0.002], as expected due to leaky integration (see Table 2). Figs 1–3 show masked threshold shifts with the equally loud short PPD and long PPD maskers at the soft, medium, and loud presentation levels, respectively.

**Fig 4:**
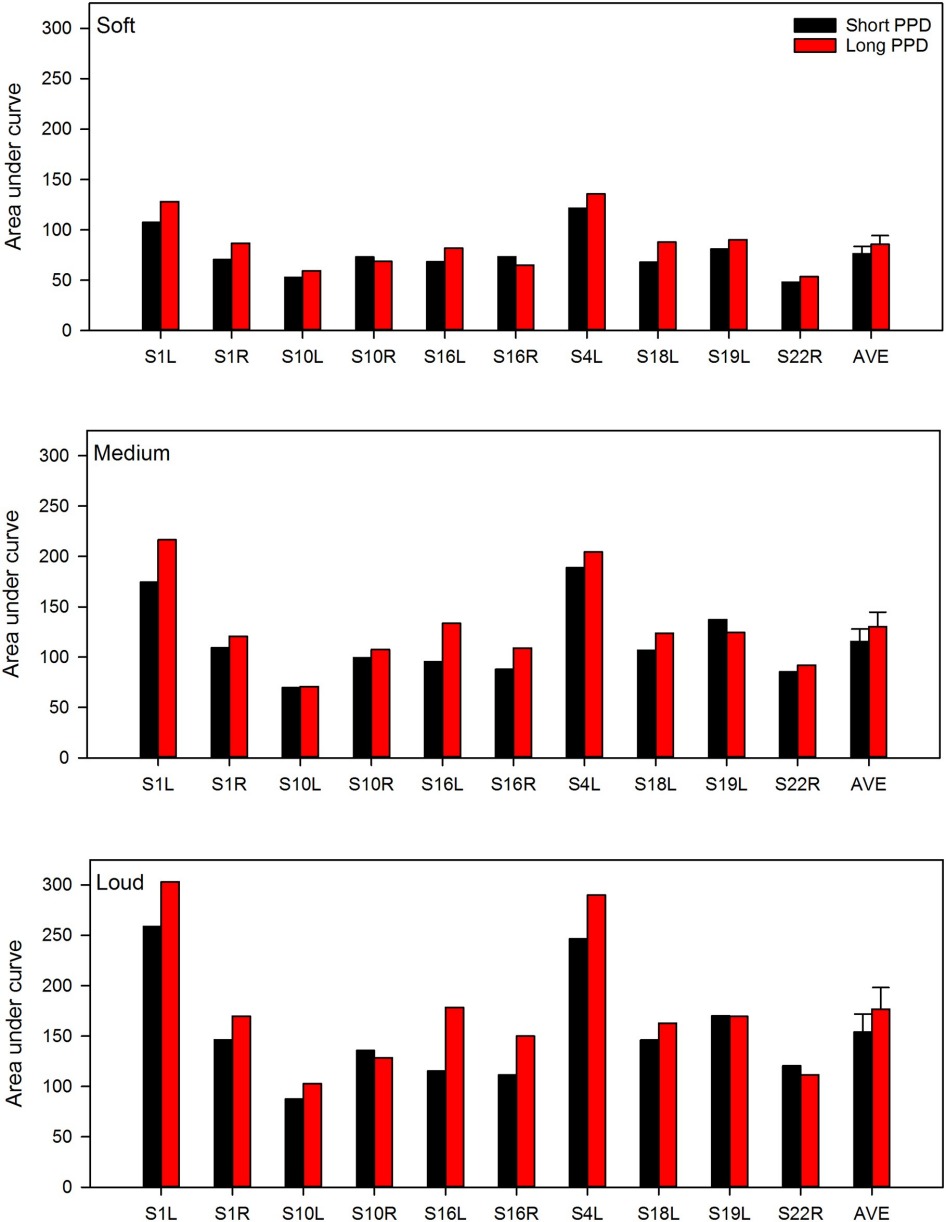
Area under the curve (AUC) for the masking functions shown in Figs 1–3. The error bars show the standard error.

Fig 4 shows individual and mean AUC values at the soft, medium, and loud masker levels for the equally loud short and long PPD maskers. A general linear model was performed on the AUC data shown in Fig 4, with masker level (soft, medium, loud) and masker type (short PPD, long PPD) as fixed factors, and test ear as the random factor. Results showed significant effects of masker level [F(2,18) = 48.3, p<0.001], masker type [F(1,9) = 11.9, p = 0.007] and test ear [F(9,22) = 11.26, p<0.001]. There was no significant interaction between masker type and masker level [F(2,18) = 2.8, p = 0.089], but there were significant interactions between masker type and test ear [F(18,18) = 8.73, p <0.001], and between masker level and test ear [F(9,18) = 3.37, p = 0.0091].

**Fig 5:**
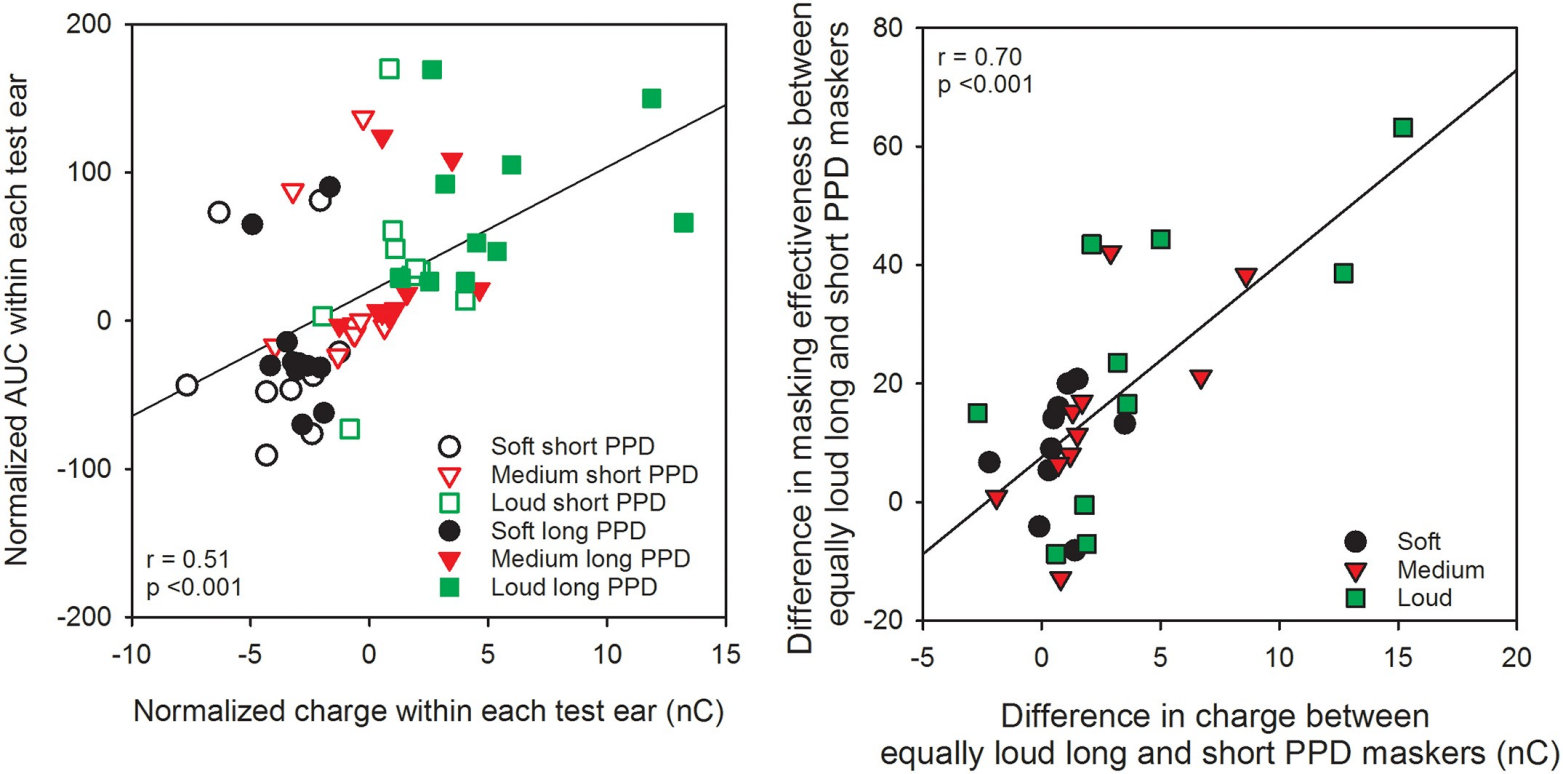
Left panel: Normalized AUC as a function of normalized charge for each test ear. Values were normalized to the mean values of each test ear. The line shows the linear regression across all data; the correlation coefficient and p value are shown at bottom left. Right panel: Difference in masking effectiveness between equally loud long and short PPD maskers, as a function of the difference in charge between the long and short PPD maskers. The line shows the linear regression across all data; the correlation coefficient and p value are shown at top right.

To test the hypothesis that the amount of masking depends on the amount of charge exerted on the cell membrane, the charge of the short and long PPD maskers was compared to AUC produced by the maskers. First, AUC and masker charge were normalized to allow comparison across test ears. The mean AUC was first calculated across the 6 AUC values (3 levels by 2 maskers) for each test ear, and then the mean AUC was subtracted from all AUC values from that test ear. Similarly, the mean masker charge was calculated and then subtracted from all charge values from that test ear. Thus, the normalization preserved the variation within test ears but removed the variation across test ears. Result showed a significant relationship between the normalized AUC and normalized masker charge (r (58) = 0.51, p < 0.001; left panel of Fig 5).

The difference in charge between the equally loud long and short PPD maskers was also compared to the difference in the masking effectiveness between the long and short PPD maskers (difference in AUC between the equally loud long and short PPD maskers); data are shown in right panel of Fig 5. A significant correlation was observed between the difference in charge and masking effectiveness of the long and short PPD maskers (r (28) = 0.70, p < 0.001). Note that at the loud presentation level, the charge difference exceeded 10 nC for test ears S16L and S16R. When these data points were removed, the correlation remained significant (r (26) = 0.54, p = 0.003).

**Fig 6:**
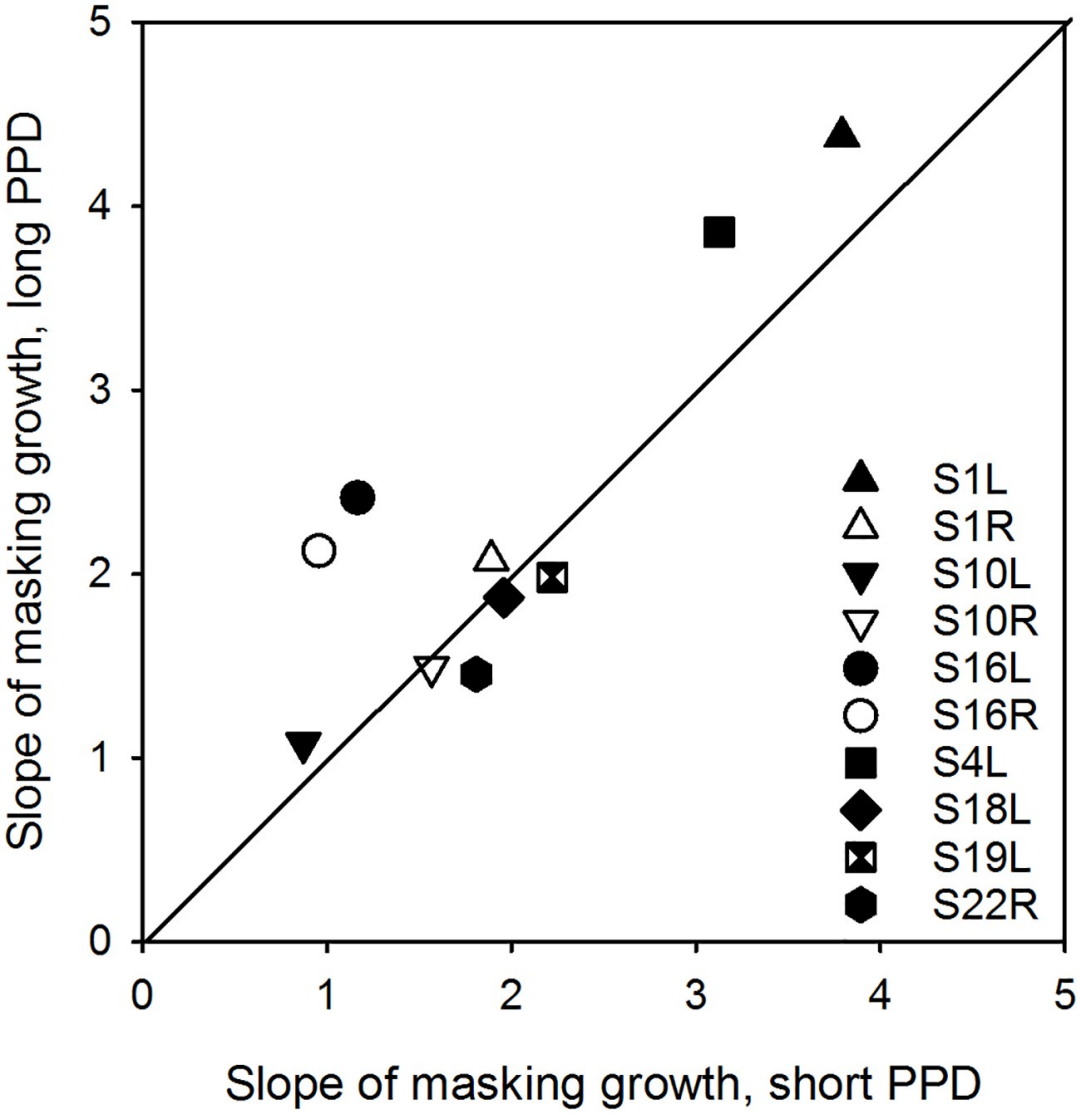
Slope of masking growth with the long PPD masker as function of slope of masking growth with the short PPD masker. Data above the diagonal line indicate steeper masking growth with the long PPD masker.

The general linear model performed on the AUC data in Fig 4 showed no interaction between masker level and type, suggesting that growth of masking was similar between the short and long PPD maskers with presentation levels. The rate of masking growth for the short and long PPD maskers was examined explicitly by fitting a linear slope between AUC values and presentation levels. Fig 6 compares the slope of masking growth with the long PPD masker with the slope of masking growth with the short PPD masker. With the exception of S16L and S16R, slopes were similar between the short and long PPD maskers (paired t-test: t(10) = -1.9, p = 0.093).

**Fig 7:**
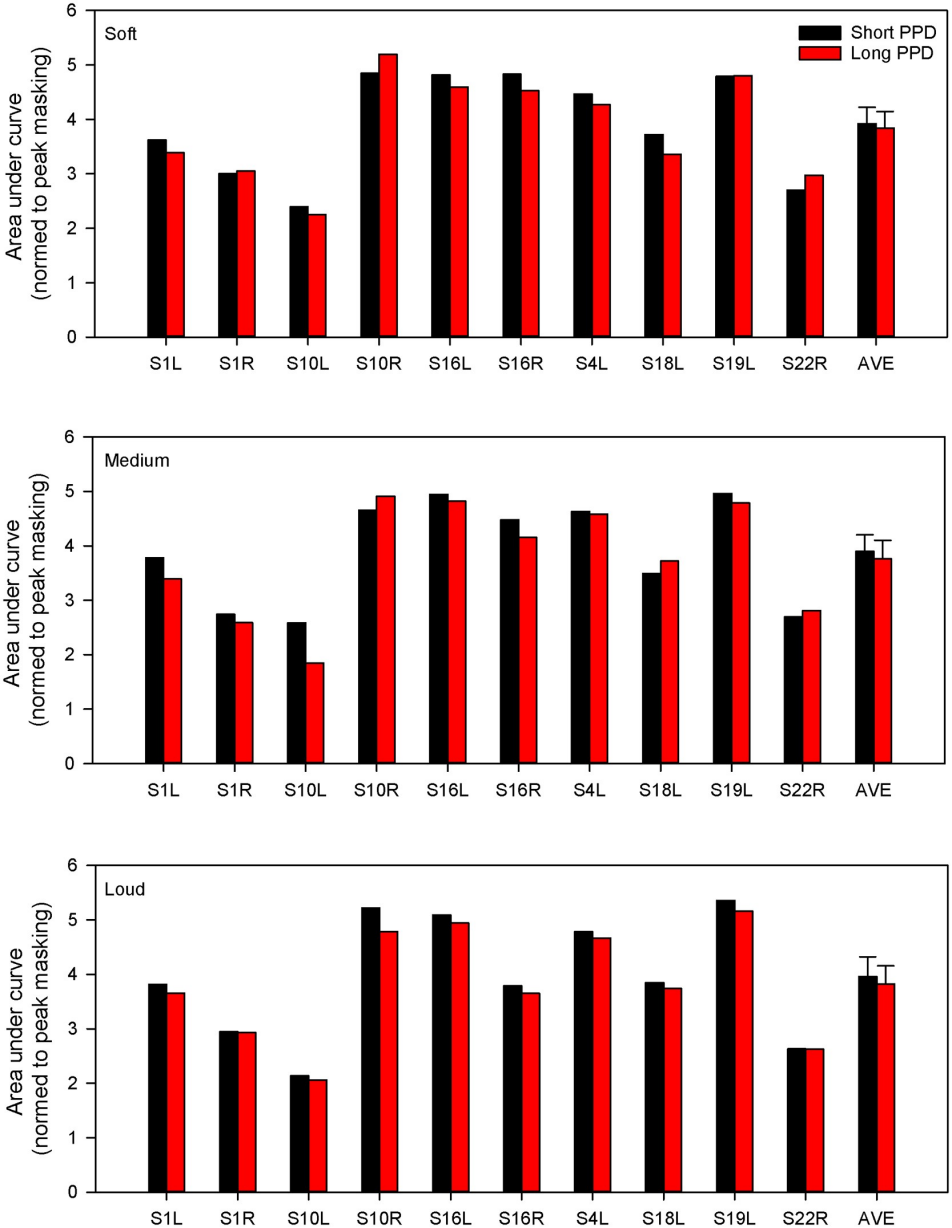
Normalized AUC with the short and long PPD maskers at the soft, medium, and loud presentation levels; threshold shifts were first normalized to the peak masking before calculating AUC. The error bars show the standard error.

Fig 7 shows the normalized AUC values (i.e., SOE) with the short and long PPD maskers at the soft, medium, and loud presentation levels; threshold shifts were normalized to the peak masking before calculating AUC. A general linear model with masker level and masker type as fixed factors and test ear as a random factor, showed that SOE was significantly smaller for the long PPD masker by 0.12 [F(1,9) = 6.9, p = 0.03]; there was no effect of masker level [F(2,18) = 0.2, p = 0.81]. There was no significant interaction between masker type and masker level [F(2,18) = 0.2, p = 0.79], or between masker type and test ear. [F(9,18) = 1.16, p = 0.37]. However, there was an interaction between masker level and test ear [F(18,18) = 3.88, p = 0.003].

## Discussion

Integration of charge on the neural membrane over the duration of a long PPD is leaky, such that the amount of charge required to achieve a given loudness can be greater with a long PPD than with a short PPD. The efficiency of charge integration depends at least partially on the condition of the auditory nerve. In clinical practice, phase duration is a parameter that is sometimes adjusted (e.g., to keep within voltage compliance, reduce facial nerve stimulation, etc.). The present study examined two perceptual consequences associated with long PPDs. First, we examined whether, for equally loud maskers, the greater charge with the long PPD would result in greater forward masking, compared to short PPD. In subsequent analysis, we evaluated differences in SOE between equally loud long and short PPD maskers. To our knowledge, these data are the first explicit comparisons between long and short PPDs in terms of overall masking and SOE.

### Masking effectiveness

For most test ears and presentation levels, the long PPD masker required a greater amount of charge to match the loudness of a short PPD masker (see Table 2). Our previous related study [5], which used the same test ears as in this study, showed that this difference in charge (defined as “charge integration efficiency”) depended on duration of hearing loss. These findings suggest that the ability of neurons to hold onto the injected charge over the duration of the pulse phase depends on the condition of the auditory neurons. Loss of peripheral processes, neurons with large fiber diameters, and reduction of neural density can all lead to greater leakiness in charge integration.

The forward masking patterns with the long and short PPD maskers were used to quantify the relationship between masker charge and masking effectiveness. This relationship was first examined with all conditions collapsed. The most obvious effect was that as presentation level (loudness) increased, the masker charge increased, and the amount of masking increased. A significant relationship was observed between masker charge and AUC for the related masking functions (left panel of Fig 5). If we assume that an increase in loudness is due to increased neural activity [33], the linear relationship is consistent with the idea that neural excitability following the forward masker is related to the masker-induced neural activity. There is abundant evidence in the physiology literature that shows that spike-rate adaptation occurs after repetitive stimulation, especially with high stimulation rates or rigorous stimulation with high current levels [13, 34–36]. The increased firing rate can have an adverse effect on the neurons’ metabolism and result in cellular energy depletion, thus reducing their excitability for subsequent stimulation. The more important question is: if maskers are equally loud and we assume that they evoke a similar amount of neural activity, would there be any difference in masking effectiveness between short and long PPDs? The present data showed that the long PPD maskers produced overall greater forward masking than did the equally loud short PPD maskers (Fig 4). This effect was also dependent on test ear. For test ears or conditions where masking effectiveness was similar between equally loud long and short PPD maskers, the maskers also had similar amounts of charge (right panel of Fig 5). Assuming again that equal loudness corresponds to equal neural activity, the present data suggest that for equally loud maskers, masking effectiveness depends on the total charge in the masker rather than masker-induced ensemble neural activity.

These results echo the findings from Zhou et al. [19], who showed that a high-rate pulse train (1000 pps) produced more masking than an equally loud low-rate pulse train (250 pps). This could be due to an accommodation effect, where sub-threshold pulses, which do not evoke action potentials in the neuron, suppress neuron responses for the subsequent pulse. This accommodation effect has been shown to be greater for high-rate than low-rate stimulation [37]. Therefore, the same neural activity evoked by two equally loud maskers of different rates, is followed by a different reduction in subsequent neural excitability. This suggests that masking effectiveness depends on characteristic of the masker, but not necessarily on the masker-induced neural response. We speculate that the greater amount of charge injected over time on the neural membrane by the longer PPD masker may not necessarily evoke more neural activity due to leakiness, but it may desensitize neurons in ways that reduce responsiveness to subsequent stimulation. This hypothesis warrants further investigation. If masking depends on the amount of charge in the masker and loudness grows more slowly with increasing charge when increasing PPD, then one would expect that masking should grow more steeply with presentation level for the long PPD masker, compared to the short PPD masker. Although this is not true for the group data, test ears S16L and S16R exhibited steeper growth of masking with increasing PPD (Fig 6); note that the PPD DR was much wider and charge integration efficiency was much poorer for these test ears, possibly due to long duration of deafness [5]. Taken together, the present data show that masking effectiveness depends on the masker’s magnitude (in charge). Therefore, for CI users that exhibit poor charge integration efficiency, greater charge will be required to establish a criterion loudness, which in turn may produce greater masking.

### Spread of excitation (SOE)

Given that the long PPD maskers would require less PA than equally loud short PPD maskers, which would produce a smaller current field, we hypothesized that SOE would be smaller with the long PPD maskers. Within the smaller current field, the long PPD masker may achieve the same amount of neural activity as the short PPD/high PA masker by increasing the firing efficiency within the smaller population of neurons. McKay and McDermott [29] reported that equally loud long and short PPD stimuli were perceived as having different pitches, supporting the idea that differences in PPD produced different spatial excitation patterns. They also reported that the CI listeners could not consistently rank the pitches, which is consistent with the idea that the center of excitation is close to the masker electrode with either a short or long PPD, and that the pitch differences were associated with differences in the spatial spread.

In agreement with our hypothesis, the present data showed that SOE was significantly smaller for the long PPD than for the short PPD. Note that effect size was rather small. Unlike the effect of PPD on masking that depended on leakiness of the ear, the effect on SOE was consistent across ears, despite the difference in the characteristics of the tested electrodes. The assumption that long PPDs excite a smaller population of neurons may offer an alternative explanation for why long PPDs produced greater masking in the present study. Because each neuron would have to be driven harder to achieve sufficient spike activity, they may be more likely to adapt. This seems unlikely, as Zhou et al. [19] showed that low-rate stimulation, which produced narrower excitation than equally loud high-rate stimulation, also produced less forward masking.

Results also showed that SOE was largely unchanged across presentation levels for both short and long PPD maskers. In acoustic hearing, SOE is narrower at lower presentation levels and tuning curves are extremely sharp due to active processes of the outer hair cells [38]. Excitation becomes broader at high presentation levels, where the passive mechanical processes dominate the excitation patterns. In electric hearing, the active processes are absent, therefore the SOE patterns are expected to remain the same at all stimulation levels. This is consistent with the previous studies that showed similar shapes of masking functions across masker levels [39], and similar tuning curve characteristics as a function of probe level [40].

### Clinical implications

It is important to note that in clinical fitting, for devices that use PPD to code intensity (e.g., Oticon), the PPD DR would depend on the level of the fixed PA, and the maximum PPD may be much smaller than the long PPDs tested here. Thus, there might not necessarily be excessive masking for PPD intensity coding, compared to PA intensity coding (which is widely used in clinical CI mapping). While not a linear tradeoff, such small PPD ranges might also require larger fixed PA values than used in the present study. For patients with severely poor charge integration efficiency (and thus a large PPD DR), using PPD intensity coding might have the detrimental effect of causing overall greater masking. For patients with good charge integration efficiency, using PPD intensity coding would not lead to greater masking, but may reduce channel interaction. The net effect of interactions among charge, masking and SOE on speech recognition remains unclear and warrants future research.

### Conclusions

In this study, forward masking patterns were compared in CI listeners for equally loud maskers with a short or long PPD; masking patterns were measured at relatively soft, medium, and loud presentation levels. Major findings include:

1. When data were collapsed across all presentation levels and masker types, forward masking increased with the amount of masker charge.
2. On average, at equal loudness, the long PPD maskers contained greater charge and produced greater overall masking than did the short PPD maskers. This suggests that masking effectiveness depends on the total charge in the masker rather than masker-induced ensemble neural activity (i.e., loudness).
3. There was a significant correlation between the difference in masking effectiveness between the long and short PPD maskers and the difference in the charge required to match loudness (charge integration efficiency).
4. While the overall masking was greater with the long PPD maskers, SOE was smaller, compared to the short PPD maskers.
5. Intensity coding with PPD may need to be optimized for CI patients according to charge integration efficiency to limit overall masking and SOE.

## Supporting information

S1 Appendix Threshold (T), maximum acceptable loudness (MAL), and dynamic range (DR) for the probe electrodes in dB (top) or microamps (bottom) for each test ear. (DOCX)
