# Supplementary material for: Effect of pulse phase duration on forward masking and spread of excitation in cochlear implant listeners
Source: PLoS One. 2020 Jul 20;15(7):e0236179. doi: 10.1371/journal.pone.0236179 (PMC7371170; doi:10.1371/journal.pone.0236179)
Supplement: S1 Appendix — (DOCX) [file pone.0236179.s001.docx]

| **El** | **8** | | | **9** | | | **10** | | | **11** | | | **12** | | | **13** | | | **14** | | |
| --- | --- | --- | --- | --- | --- | --- | --- | --- | --- | --- | --- | --- | --- | --- | --- | --- | --- | --- | --- | --- | --- |
| dB | T | MAL | DR | T | MAL | DR | T | MAL | DR | T | MAL | DR | T | MAL | DR | T | MAL | DR | T | MAL | DR |
| S1L | 38.5 | 49.0 | 10.5 | 39.0 | 49.7 | 10.8 | 40.4 | 49.7 | 9.4 | 39.3 | 48.5 | 9.2 | 40.8 | 49.9 | 9.1 | 40.2 | 49.6 | 9.4 | 41.0 | 49.0 | 8.1 |
| S1R | 31.5 | 46.6 | 15.1 | 32.1 | 46.9 | 14.9 | 34.0 | 46.4 | 12.4 | 35.6 | 45.9 | 10.2 | 34.9 | 46.7 | 11.8 | 35.9 | 46.6 | 10.7 | 36.7 | 46.6 | 9.9 |
| S10L |  |  |  |  |  |  |  |  |  | 38.9 | 45.5 | 6.6 | 38.2 | 46.6 | 8.3 | 38.7 | 46.7 | 8.0 | 39.2 | 46.6 | 7.3 |
| S10R | 28.5 | 43.0 | 14.6 | 28.3 | 43.9 | 15.6 | 29.3 | 44.5 | 15.2 | 30.4 | 45.3 | 15.0 | 31.1 | 45.5 | 14.4 | 32.2 | 45.9 | 13.6 | 32.0 | 44.6 | 12.7 |
| S16L | 38.5 | 55.5 | 17.0 | 39.2 | 55.0 | 15.8 | 37.2 | 54.0 | 16.7 | 37.1 | 53.4 | 16.3 | 35.1 | 53.2 | 18.1 | 34.9 | 54.3 | 19.4 | 34.1 | 53.8 | 19.7 |
| S16R | 37.3 | 56.9 | 19.7 | 37.1 | 55.0 | 17.9 | 37.7 | 57.5 | 19.8 | 37.1 | 55.2 | 18.1 | 36.4 | 55.2 | 18.8 | 36.2 | 57.5 | 21.3 | 37.1 | 56.2 | 19.2 |
| S4L | 29.7 | 41.6 | 11.9 | 29.5 | 40.9 | 11.5 | 29.9 | 41.5 | 11.6 | 30.4 | 40.6 | 10.2 | 29.8 | 39.4 | 9.5 | 30.5 | 39.9 | 9.4 | 30.3 | 39.9 | 9.6 |
| S18L | 29.5 | 44.5 | 15.0 | 31.5 | 45.2 | 13.7 | 32.0 | 44.3 | 12.3 | 29.8 | 44.1 | 14.3 | 31.2 | 45.2 | 13.9 | 31.0 | 43.6 | 12.6 | 30.7 | 43.6 | 12.9 |
| S19L | 30.3 | 41.8 | 11.5 | 30.4 | 40.9 | 10.5 | 29.8 | 41.5 | 11.6 | 29.7 | 41.5 | 11.8 | 29.2 | 41.6 | 12.5 | 29.6 | 41.1 | 11.5 | 28.7 | 40.8 | 12.0 |
| S22L | 31.1 | 45.9 | 14.8 | 29.5 | 45.7 | 16.2 | 28.6 | 45.3 | 16.7 | 30.5 | 41.6 | 11.1 | 30.9 | 44.1 | 13.2 | 32.4 | 45.3 | 12.9 | 31.8 | 45.5 | 13.8 |

| **EL** | **8** | | | **9** | | | **10** | | | **11** | | | **12** | | | **13** | | | **14** | | |
| --- | --- | --- | --- | --- | --- | --- | --- | --- | --- | --- | --- | --- | --- | --- | --- | --- | --- | --- | --- | --- | --- |
| μA | T | MAL | DR | T | MAL | DR | T | MAL | DR | T | MAL | DR | T | MAL | DR | T | MAL | DR | T | MAL | DR |
| S1L | 84 | 283 | 199 | 89 | 307 | 218 | 104 | 307 | 202 | 92 | 266 | 174 | 109 | 313 | 204 | 102 | 300 | 198 | 112 | 283 | 171 |
| S1R | 37 | 213 | 175 | 40 | 222 | 182 | 50 | 209 | 159 | 60 | 196 | 136 | 56 | 217 | 162 | 62 | 213 | 151 | 68 | 213 | 145 |
| S10L |  |  |  |  |  |  |  |  |  | 88 | 189 | 100 | 82 | 213 | 131 | 86 | 217 | 131 | 91 | 213 | 122 |
| S10R | 26 | 142 | 116 | 26 | 157 | 131 | 29 | 167 | 138 | 33 | 185 | 152 | 36 | 189 | 153 | 41 | 196 | 156 | 40 | 170 | 131 |
| S16L | 84 | 598 | 514 | 91 | 563 | 472 | 73 | 499 | 426 | 71 | 469 | 398 | 57 | 460 | 403 | 56 | 519 | 464 | 51 | 489 | 438 |
| S16R | 73 | 703 | 631 | 71 | 563 | 492 | 77 | 747 | 671 | 72 | 574 | 503 | 66 | 574 | 508 | 64 | 747 | 683 | 71 | 649 | 577 |
| S4L | 31 | 121 | 90 | 30 | 111 | 82 | 31 | 118 | 87 | 33 | 107 | 74 | 31 | 93 | 62 | 33 | 99 | 65 | 33 | 99 | 66 |
| S18L | 30 | 167 | 137 | 37 | 181 | 144 | 40 | 164 | 124 | 31 | 160 | 129 | 36 | 181 | 145 | 35 | 151 | 116 | 34 | 151 | 117 |
| S19L | 33 | 123 | 91 | 33 | 111 | 78 | 31 | 118 | 87 | 31 | 118 | 88 | 29 | 121 | 92 | 30 | 114 | 83 | 27 | 109 | 82 |
| S22L | 36 | 196 | 161 | 30 | 192 | 163 | 27 | 185 | 158 | 34 | 121 | 87 | 35 | 160 | 125 | 42 | 185 | 143 | 39 | 189 | 150 |

S1 Appendix. Threshold (T), maximum acceptable loudness (MAL), and dynamic range (DR) for the probe electrodes in dB (top) or microamps (bottom) for each test ear.
